# Systematic Characterization of *TCP* Gene Family in Four Cotton Species Revealed That *GhTCP62* Regulates Branching in *Arabidopsis*

**DOI:** 10.3390/biology10111104

**Published:** 2021-10-26

**Authors:** Zhao Liu, Jingyu Yang, Shengdong Li, Le Liu, Ghulam Qanmber, Guoquan Chen, Zhenyu Duan, Na Zhao, Gang Wang

**Affiliations:** 1 Zhengzhou Research Base, State Key Laboratory of Cotton Biology, Zhengzhou University, Zhengzhou 450001, Henan, China; liuzhaocaas@zzu.edu.cn (Z.L.); yjyhzz@163.com (J.Y.); lllsd@gs.zzu.edu.cn (S.L.); liule@gs.zzu.edu.cn (L.L.); cgq@gs.zzu.edu.cn (G.C.); 2 State Key Laboratory of Cotton Biology, Institute of Cotton Research, Chinese Academy of Agricultural Sciences, Anyang 455000, Henan, China; gqkhan12@gmail.com; 3 Xinjiang Academy of Agricultural and Reclamation Science, Shihezi 832000, Xinjiang, China; dzy19900451@126.com

**Keywords:** *TCP*, plant architecture, *GhTCP62*, shoot branching, cotton, *Arabidopsis thaliana*

## Abstract

**Simple Summary:**

*TCP* transcription factors (TF) are indispensable for the normal functioning of plant growth and development. This study identified and performed phylogenetic analysis on 218 *TCP* genes in four cotton species. We also observed conserved exon-intron structures and protein motif distribution patterns in *GhTCP* genes. *GhTCP62* was enriched in the axillary bud, indicating that it plays a role in branching. *GhTCP62* is a nuclear-localized TF, the overexpression of which decreases the number of cauline-leaf branches and rosette-leaf branches in *Arabidopsis*. Additionally, the expression levels of *HB21* and *HB40* genes increased in plants with *GhTCP62* overexpression, demonstrating that *GhTCP62* could regulate branching by regulating *HB21* and *HB40*. Collectively, the *GhTCP62* TF located in the nucleus was highly enriched in the axillary buds, and *GhTCP62* overexpression lines demonstrated fewer rosette-leaf branches and cauline-leaf branches, indicating that *GhTCP62* regulates branching in *Arabidopsis*.

**Abstract:**

TEOSINTE-BRANCHED1/CYCLOIDEA/PCF *(TCP*) transcription factors play an essential role in regulating various physiological and biochemical functions during plant growth. However, the function of *TCP* transcription factors in *G. hirsutum* has not yet been studied. In this study, we performed genome-wide identification and correlation analysis of the *TCP* transcription factor family in *G. hirsutum*. We identified 72 non-redundant *GhTCP* genes and divided them into seven subfamilies, based on phylogenetic analysis. Most *GhTCP* genes in the same subfamily displayed similar exon and intron structures and featured highly conserved motif structures in their subfamily. Additionally, the pattern of chromosomal distribution demonstrated that *GhTCP* genes were unevenly distributed on 24 out of 26 chromosomes, and that fragment replication was the main replication event of *GhTCP* genes. In *TB1* sub-family genes, *GhTCP62* was highly expressed in the axillary buds, suggesting that *GhTCP62* significantly affected cotton branching. Additionally, subcellular localization results indicated that *GhTCP62* is located in the nucleus and possesses typical transcription factor characteristics. The overexpression of *GhTCP62* in *Arabidopsis* resulted in fewer rosette-leaf branches and cauline-leaf branches. Furthermore, the increased expression of *HB21* and *HB40* genes in *Arabidopsis* plants overexpressing *GhTCP62* suggests that *GhTCP62* may regulate branching by positively regulating *HB21* and *HB40*.

## 1. Introduction

The structure of the aerial parts of plants plays a decisive role in crop development and yield as well as in photosynthesis [1,2]. For instance, wheat varieties with short and sturdy stems produce higher yields and harvest efficiency and are more resistant to lodging [3]. The yield of soybean varieties differs due to differences in branching plasticity [4,5]. In the case of maize (*Zea mays*), plants with an upright structure are better suited for intensive planting [6]. Plant architecture is determined by the position and differentiation of the meristem, which is reflected in plant organs such as stems and branches. The shoot apical meristem (SAM) affects plant elongation and axillary meristems (AMs) determine lateral branching, which ultimately alters the shoots’ architecture [7,8]. So far, studies have shown that the biological effects of plant structure can be regulated by changing gene expression, in which transcription factors (TFs) play a surprising role [9,10]. The overexpression of *GmMYB14* in soybeans leads to a decrease in endogenous Brassinosteroid (BR) content and a semi-dwarf and compact plant structure, which improves yield [11]. Similarly, the overexpression of *AtDREB1B* caused a significant decrease in the quantitative and morphological traits in *Arabidopsis*, particularly plant height [12]. The expression of the tomato *WRKY* gene *SlWRKY23* in transgenic *Arabidopsis* displayed a host of branching in inflorescences [13]. The gain-of-function mutant *exb1-D* enhances *WRKY71* gene expression and produces a dense and stunted phenotype [14].

The plant-specific transcription factor family TEOSINTE-BRANCHED1/CYCLOIDEA/PCF (*TCP*) has been known to regulate the development of plant branches in many species [15,16]. These transcription factors share a conserved domain consisting of 59 amino acids at the N-terminus, which is known as the *TCP* domain. This domain was initially identified in four proteins encoded by apparently unrelated genes, which were subsequently named "*TCP*": *TEOSINTE BRANCHED1 (TB1)* in maize (*Zea mays*), *CYCLOIDEA* (*CYC*) in snapdragon (*Antirrhinum majus*), and *PROLIFERATING CELL NUCLEAR ANTIGEN FACTOR1* (*PCF1*) and *PROLIFERATING CELL NUCLEAR ANTIGEN FACTOR2* (*PCF2*) in rice (*Oryza sativa*). *TB1* is involved in regulating the growth inhibition of the formation of axillary buds on the lateral branches [17,18], *CYC* is expressed in early lateral flowering regions and regulates the symmetrical development of flowers [19], and *PCF1* and *PCF2* bind to the promoter of the rice *PCNA* gene and help regulate the cell cycle [20]. *TCP* proteins are grouped into two subfamilies: class I can bind GGNCCCAC elements and mainly induces cell division; class II can bind GTGGNCCC elements and inhibits growth and development [21,22]. Additionally, class Ⅱ is further divided into the CINCINNATA (CIN) and CYC/TB1 subfamilies, based on differences in *TCP* domain sequences [23,24]. As genome technologies have advanced, more and more plant species with *TCP* family genes have been identified, including 24 members of the *TCP* transcription factor family in *Arabidopsis* [25]; 22 *TCP*s in rice (*Oryza sativa*) [26]; 36 *TCP*s in the tomato genome [27]; 46 *ZmTCP* genes in maize (*Zea mays L.*) [28]; 17 *TCP*s in the leaf transcriptome of tea tree (*C. sinensis*) [29]; 22 and 20 *TCP*s in wild (*Hordeum vulgare subsp. spontaneum, Hs*) and cultivated barley (*Hordeum vulgare subsp. vulgare, Hv*), respectively [30]; 42 *TCP*s in switchgrass (*Panicum virgatum L.*) [31]; and 66 *TCP*s in wheat (*Triticum aestivum L.*) [32]. Recent studies have analyzed *TCP* functionality and found the following: decreases in *AtTCP*2 and *AtTCP4* transcripts regulate large and crinkly leaves (called the JAW phenotype); the overexpression of *AtTCP4* leads to early maturity and smaller leaves in *Arabidopsis* [33]; when the expression levels of the triple T-DNA insertion mutant *tcp5/13/17* are significantly knocked down, there is a delay in the flowering phenotype [34].

Cotton is an important global crop and produces valuable natural fibers. *Gossypium hirsutum* is an allotetraploid, which consists of A gene subgroup (At) and D gene subgroup (Dt). The A gene subgroup originates from *Gossypium arboreum*, and the D gene subgroup originates from *Gossypium raimondii* [35]. Recently, *TCP* genes have been identified and analyzed in several cotton varieties: 38 and 36 *TCP*s were identified in the genome of the diploid cotton species *Gossypium raimondii* and *Gossypium arboreum*, respectively, while 75 *TCP* genes were identified in sea-island cotton (*Gossypium barbadense*) and 73 in upland cotton (*Gossypium hirsutum L.*) TM-1 genome [36,37]. In cotton, *GbTCP* promotes fiber elongation by regulating the content of endogenous JA [38]. *GhCUC2* and *GhTIE1* activate the transcriptional activity of *GhBRC1* and inhibit branch development through ABA signaling [39]. *GhTCP21* and *GhTCP54* both responded to salt and drought stress [37]. *GhTCP14* regulates auxin response, while the expression of transporter genes affects fiber differentiation and elongation [40]. While there has been significant research on how *TCP* transcription factors affect plant development, few studies have assessed the mechanism affecting the architecture of cotton plants.

In our study, 72 *GhTCP* family genes were systematically identified and analyzed, the first such genome-wide characterization in the upland cotton (*Gossypium hirsutum L.*) ZM24 genome. We also performed phylogenetic analysis, genomic structures, and conserved motifs analysis, sequences logo analysis for conserved amino acid residues, chromosomal location analysis, and synteny analysis. Next, we analyzed the TB1 clade, which can significantly affect the development of plant branches. We initially studied the tissue-specific expression profile of TB1clade members and then determined the subcellular localization of *GhTCP62* in the leaf epidermal cells of *Nicotiana benthamiana*. We then overexpressed *GhTCP62* to explore how it relates to branching in *Arabidopsis*. Our study provides insight into how *TCP* regulates branch development in *Arabidopsis* and lays the foundation for subsequently creating an ideal plant architecture suitable for intensive planting and mechanized harvesting.

## 2. Materials and Methods

### 2.1. Sequence Retrieval and Information Statistics of TCP Proteins

First, we downloaded genes containing the *TCP* domain in *Arabidopsis* from TAIR 10 (http://www.Arabidopsis.org, accessed on 30 March 2021). We then retrieved the *Arabidopsis TCP* genes using a query to identify *TCP* genes in *Gossypium hirsutum*, *Gossypium raimondii*, *Gossypium arboreum*, and *Gossypium barbadense.* The *G. hirsutum* and *G. raimondii* genome sequences were downloaded from the cotton functional genomics database (http://grand.cricaas.com.cn/home, accessed on 30 March 2021), and the *G. arboreum* and *G. barbadense* database genome sequences were downloaded from COTTONGEN (https://www.cottongen.org/, accessed on 30 March 2021). Next, the conserved domains of the proteins encoded by the homologous genes of *G. hirsutum*, *G. raimondii*, *G. arboreum*, and *G. barbadense* were analyzed using the NCBI Batch CD-Search (https://www.ncbi.nlm.nih.gov/Structure/bwrpsb/bwrpsb.cgi, accessed on 30 March 2021), batch SMART (http://smart.embl-heidelberg.de/smart/batch.pl, accessed on 30 March 2021), and Pfam (http://pfam.xfam.org/search#tabview=tab1, accessed on 30 March 2021). We then renamed the *TCP* gene identified in four cotton species according to the position of the genes on the chromosomes. Furthermore, we located the chromosome of the *TCP* gene in *G. hirsutum* using the annotation file of its genome.

### 2.2. Phylogenetic Analysis, Gene Structure and Conserved Motif

We performed multiple sequence alignments (MSA) of *TCP* proteins in *Arabidopsis*, *G. hirsutum*, *G. raimondii*, *G. arboreum*, and *G. barbadense* genomes using Muscle, with default settings [41]. Next, we constructed a rooted evolutionary tree using the Neighbor-Joining (NJ) method [42]. Finally, we constructed a separate phylogenetic tree containing all the *GhTCP* protein sequences for subsequent analysis.

The exons-introns coordinates of the genes were extracted from the genome gene annotation results, and the conserved motifs were predicted using the online MEME program (http://meme-suite.org/tools/meme, accessed on 30 March 2021) [43], with default parameters. TBtools was used to display the gene structure and conserved motifs along with the phylogenetic tree [44].

### 2.3. Analyses of Chromosomal Distribution and Collinearity

The *G. hirsutum* genome annotation file (https://cottonfgd.org/about/download.html, accessed on 30 March 2021) was used to determine the chromosomal location of the Gh*TCP* genes, after which a gff3-file was extracted. To map the physical location of the *GhTCP* genes, we used TBtools software to visualize the distribution of the *TCP* genes on the relevant chromosomes. For collinearity analysis, a collinearity module was generated for the *TCP* gene in *G. hirsutum* and *Arabidopsis*. We used these results to construct a collinearity map of the genes using CIRCOS software [45].

### 2.4. Plant Materials and Growth Conditions

In this study, the *Arabidopsis* ecotype Columbia-0 (Col-0) was used as the wild-type (WT) and for the ectopic transformation of the *GhTCP62* gene. The *Arabidopsis* seeds were disinfected with 75% alcohol and rinsed five times with sterile water. We placed the *Arabidopsis* seeds in a refrigerator at 4 °C for 72 h, in the dark, to vernalize the seeds. Next, we spread the seeds evenly on the Murashige and Skoog (MS) medium. The seedlings were grown at a constant temperature of 18–22 °C and under a 16 h light/8 h dark photoperiod, as previously described [46]. After seven days, the seedlings were transferred into pots containing a mixture of vegetative soil and vermiculite (*v/v* = 2/1) and grown at 18–22 °C under long-day conditions (16 h light and 8 h dark, 70% relative humidity). The *Arabidopsis* plants were grown in the growth chamber for a month and then used for transformation, as previously described [47,48,49].

The cotton material used in this study was ZM24, which is a variety of *G. hirsutum*. The cotton seeds were soaked in sterile water for 24 h before they were planted in a mixture of vegetative soil and vermiculite (*v/v* = 2/1). Subsequently, the cotton was grown with regular watering at 27/20 °C, 14/10 h of regulated conditions, and 75% humidity.

We also used tobacco for the subcellular localization experiments. First, we soaked the tobacco seeds in sterile water for 24 h. The seeds were then planted in pots containing a mixture of vegetative soil and vermiculite (*v/v* = 2/1). The tobacco was planted and regularly watered for two weeks at 27/20 °C, 14/10 h, and 75% humidity.

### 2.5. Gene Expression Assays

The cotton seedlings grew at 28 °C, with 16 h of light and 8 h of darkness, and were watered once every three days; after two months of growth, roots, stems, leaves, flowers, ovules, fibers, axillary bud and phyllophore were taken; and after flowering, fibers and ovules with 5, 10, 15, 20 and 25DPA are taken and immediately put into liquid nitrogen for preservation. For *Arabidopsis*, we obtained a rosette disc with a part of the stem to analyze the expression pattern. We then extracted the RNA using an RNAkey™ Reagent (SM129-02, Sevenbio, Beijing, China). An All-in-one First Strand cDNA Synthesis Kit Ⅲ for qPCR (with dsDNase) (SM135-01, Sevenbio, Beijing, China) was used to extract the cDNAs, as previously described [50,51,52].

For the RT-qPCR, the following parameters were used: 94 °C for 30 s, 40 cycles at 94 °C for 5 s, 60 °C for 15 s, and 72 °C for 10 s on a LightCycler 480 II qRT-PCR System (Applied Biosystems, Thermo Fisher Scientific, Waltham, MA, USA). Diluted cDNA was used for the RT-qPCR with SYBR Premix Ex Taq (Tli RNaseH Plus, Takara, Dalian, China), while *GhUBQ7* (accession No. DQ116441) was used as an internal control. We checked the dissociation curves of each reaction and used the cycle threshold (CT) 2^−∆∆Ct^ method to calculate the expression level of each target gene [53], as previously described [54,55]. For the relative expression level, we take the root as the standard, and set its expression level as 1. The expression of genes in other tissues refers to the expression in the root. A minimum of three biological replicates was performed for each reaction. All the primers used in the real-time quantitative RT-PCR are listed in Table S3.

### 2.6. Construction of Overexpression Vector and Subcellular Localization Vector

All the vectors used in this study were constructed by one-step cloning. This method entails using primers with homologous arms and double restriction sites to amplify gene fragments, after which vectors with the same homologous arms and restriction sites are linked to the amplified gene fragments. We then attached these vectors with the *Agrobacterium* strain GV3101 using the freeze-thaw method.

For the *GhTCP62* overexpression lines (OE), the *GhTCP62* encoding region was amplified from full-length cDNA using *GhTCP62*-specific primers. Using the 35 s promoter of the Cauliflower mosaic virus, the full-length coding region of *GhTCP62* was cloned into the EcoRI and KpnI enzyme sites of the pCAMBIA2300-GFP vector to produce the 35S::*GhTCP62* construct, which was then attached to the *Agrobacterium* strain GV3101. The *Arabidopsis* transformation was performed via the floral dip method [56]. Transgenic plants were selected on an MS medium containing 50 µg·L^−1^ kanamycin. The primers used for the vector construction method are listed in Table S3.

For the subcellular localization of *GhTCP62*, we cloned the *GhTCP62* encoding region into the pCAMBIA2300-YFP vector and fused it with the *Agrobacterium* strain GV3101. The *Agrobacterium* strain GV3101 was introduced into tobacco leaves via infiltration to detect transient expression. The tobacco plants were grown in the dark for 16 h and in light for 24–36 h. Finally, the YFP signal was detected using a confocal microscope.

## 3. Results

### 3.1. Identification of TCP Gene Family in G. hirsutum

For the *Arabidopsis TCP* family genes (Table S1), we identified the *TCP* family genes in *G. hirsutum* using NCBI Batch CD-Search, Batch SMART, and Pfam. We identified a total of 72 *GhTCP* genes in *G. hirsutum*. The *GhTCP* genes in *G. hirsutum* were named according to their position on the chromosomes, ranging from *GhTCP1* to *GhTCP72* (Table S2). We analyzed basic information about the *TCP* genes in *G. hirsutum* and *Arabidopsis* and found that the length of the amino acids of the 72 *GhTCP* transcription factors ranged from 196 (*GhTCP72*) to 550 (*GhTCP2*) amino acids, with an average of 357 amino acids. We also analyzed the basic information of the *TCP* family genes in *Arabidopsis*, and the results showed that the identified *GhTCP* genes had a similar coding length to that of *Arabidopsis*. We also observed the chromosomal positions of the *GhTCP* genes (Table S2).

### 3.2. Phylogenetic Analysis of TCP Gene Family

**Figure 1:**
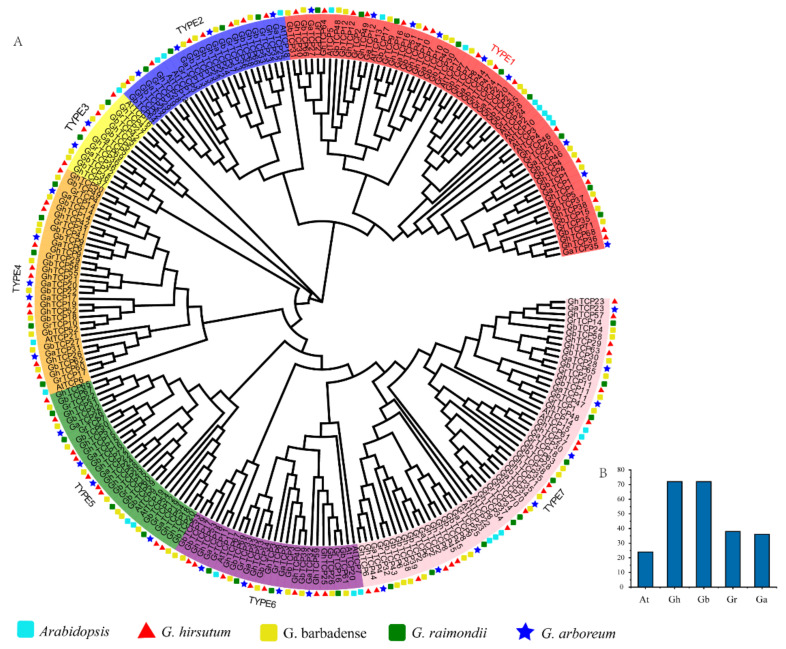
Phylogenetic analysis of *TCP* gene family members from *Arabidopsis, G. hirsutum, G. barbadense, G. raimondii*, and *G. arboreum*. (**A**) An unrooted phylogenetic tree was constructed using MEGA 6.0 and the Neighbor-Joining method, while the bootstrap test was performed with 1000 iterations. The seven subclades are indicated using different colors; TYPE1 belongs to the CIN protein, TYPE2 belongs to the TB1 protein, and TYPE3–7 belongs to the PCF protein. (**B**) Statistics of the number of *TCP* family genes in different species.

To explore the evolutionary history and phylogenetic relationship of the *GhTCP* genes, we identified 38 *GrTCPs* in *G. raimondii*, 36 *GaTCPs* in *G. arboreum*, and 72 *GbTCPs* in *G. barbadense*. Coupled with 23 *AtTCPs* and 72 *GhTCPs*, using the Neighbor-Joining method, a phylogenetic tree was constructed (Figure 1). According to the phylogenetic tree, the *TCP* gene family can be grouped into seven subfamilies, from TYPE1 to TYPE7. According to the sequence characteristics of the conserved domain of the *TCP* genes, the identified *TCP* genes can be further divided into two types: TYPE 1 and TYPE 2 belong to the second subfamily and the rest belong to the first subfamily [57]. Among them, TYPE1 (CIN) is the largest evolutionary branch, with 60 members, accounting for 24.8% of the total *TCP* proteins. TYPE3 is the smallest evolutionary branch, with only 13 members, accounting for 5.4% of total *TCP* proteins. Overall, the *TCP* protein family was sparsely distributed in different branches, indicating that the *TCP* protein family expanded before the lineage differentiation. In addition, the *TCP* proteins were unevenly distributed in some branches of the phylogenetic tree. Many *TCP* proteins in *Arabidopsis* had two or more counterparts in four cotton species, indicating that replication events occurred in the *TCP* proteins after differentiation in four cotton species and *Arabidopsis*.

Many *TCP* proteins with similar functions in *Arabidopsis* are clustered on the same branch, indicating that the *TCP* proteins of cotton on the same branch could also have similar functions. For instance, in the subfamily of TYPE1, several studies have demonstrated the redundant role of eight *CIN* proteins in lateral organ organogenesis, which interfere with several cellular pathways that control leaf development [58,59,60]. The TYPE1 subfamily contains eight *AtTCP* proteins, 16 *GhTCP* proteins, 18 *GbTCP* proteins, nine *GaTCP* proteins, and nine *GrTCP* proteins. In TYPE 2, *AtTCP1*, *AtTCP12* (BRANCHED2), and *AtTCP18* (BRANCHED1) all belong to the *TB1* protein, which plays a role in the formation of collateral and determines bud structure [61]. Bioinformatics analysis indicated that seven *GhTCP* proteins, seven *GbTCP* proteins, four *GaTCP* proteins, and four *GrTCP* proteins in a subfamily belong to the *TB1* protein, indicating that these genes also play a role in the development of collateral branches. Additionally, in the TYPE7 group, there are 5 *AtTCP* proteins, 18 *GhTCP* proteins, 16 *GbTCP* proteins, 8 *GaTCP* proteins, and 10 *GrTCP* proteins clustered together, indicating that they may perform similar functions.

### 3.3. Gene Structure and Conserved Motifs

**Figure 2:**
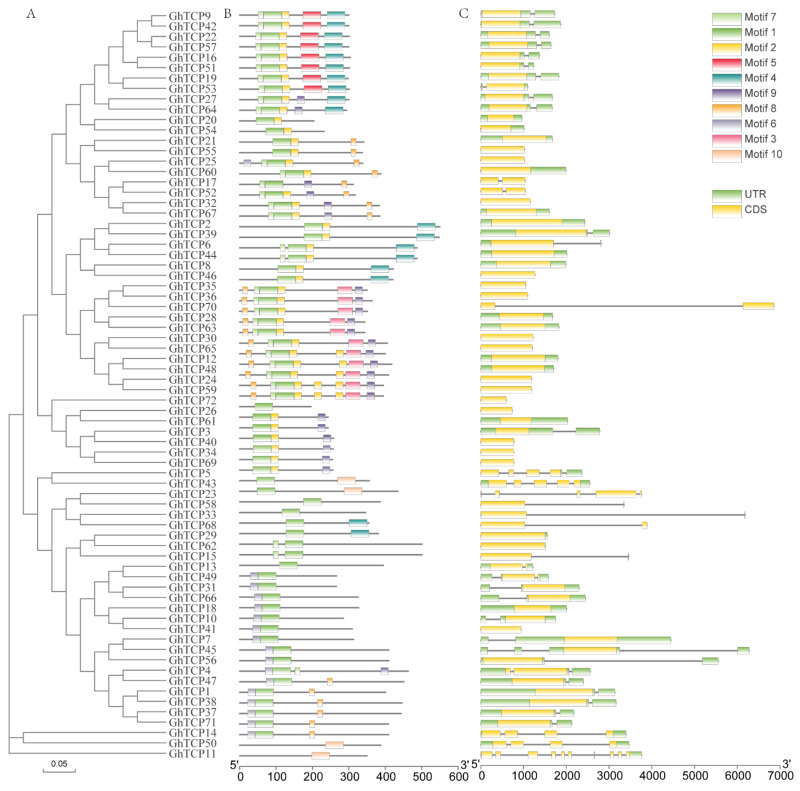
Phylogenetic analysis, exon-intron organization, and motif compositions of *G. hirsutum TCP* genes. (**A**) An evolutionary tree of all *TCP* transcription factors in *G. hirsutum* was constructed using the Neighbor-Joining method, and bootstrapped tests were performed with 1000 iterations. (**B**) Identification of conserved protein motifs in the *TCP* family was performed using the MEME program. Each pattern has a distinctive color. (**C**) Exon-intron organization of the *TCP* genes of *G. hirsutum* (ZM24). The green boxes represent exons and black lines indicate introns.

To further understand the *TCP* family genes, we obtained the exon/intron structure of each gene from the genome annotation file (Figure 2C). As a result, we found that 55 out of 72 *GhTCP* genes have no introns, while the other *GhTCP* genes typically only have one intron; only three *GhTCP* genes contain more than three introns. Introns play an important role in the evolution of different plant species, and newly evolved species may possess fewer introns than their ancestors [62]. Most *GhTCP* family genes contain only one intron, which indicates that the *GhTCP* gene family could have appeared in early evolutionary stages and subsequently expanded during later stages. Additionally, according to the evolutionary tree and gene structure, most genes in the same subfamily show extremely high similarities in exon length and number of introns (Figure 2A).

Next, we performed a conserved motif analysis of the *GhTCP* proteins using MEME software to observe their diversity of motif composition (Figure 2B). We identified conserved motifs from a total of 10 *GhTCP* proteins: motifs 1 through 10. Of these, motif 1 is a *TCP* conserved domain and is found in all *GhTCP* proteins. Almost all the *GhTCP* proteins in the same branch of the evolutionary tree possess a similar motif composition, while significant differences can be seen in different branches, indicating that *GhTCP* members in the same branch could play similar roles and that some motifs could play important roles. However, some motifs only exist in specific branches, indicating that the genes possessing these motifs may perform special functions. In general, the motif composition of most *GhTCP* proteins and the consistency of the exon/intron structure of *GhTCP* genes within the phylogenetic subfamilies further indicates that there is a close evolutionary relationship between *GhTCP* genes and the reliability of systematic analysis.

**Figure 3:**
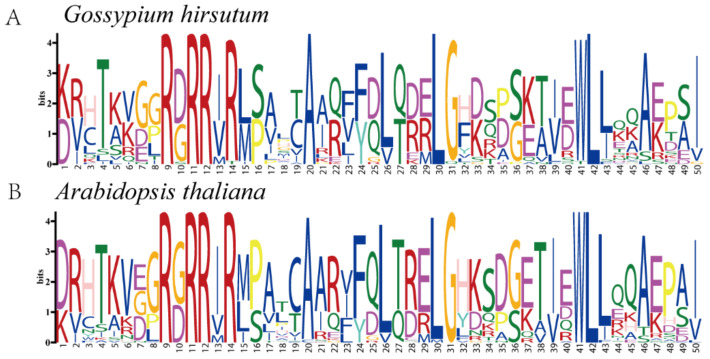
Sequence logos for conserved amino acid residues in (**A**) *G. hirsutum* and (**B**) *Arabidopsis*.

To further explore whether the *TCP* family of proteins was conserved during evolution, sequence markers were generated for conserved amino acid residues in *G. hirsutum* and *Arabidopsis* (Figure 3). Analysis of the conserved amino acid residues demonstrated that the sequence markers between the two species were markedly conserved throughout the sequence. For instance, the amino acid residues T (4), R (9), R (11), R (14), A (20), F (24), L (26), G (31), W (41), L (42), L (43), A (46), and I (50) were highly conserved between *Arabidopsis* and *G. hirsutum*. These results suggest that the *TCP* family in *G. hirsutum* and *Arabidopsis* may perform similar functions.

### 3.4. Chromosomal Distribution and Gene Collinearity Analysis

**Figure 4:**
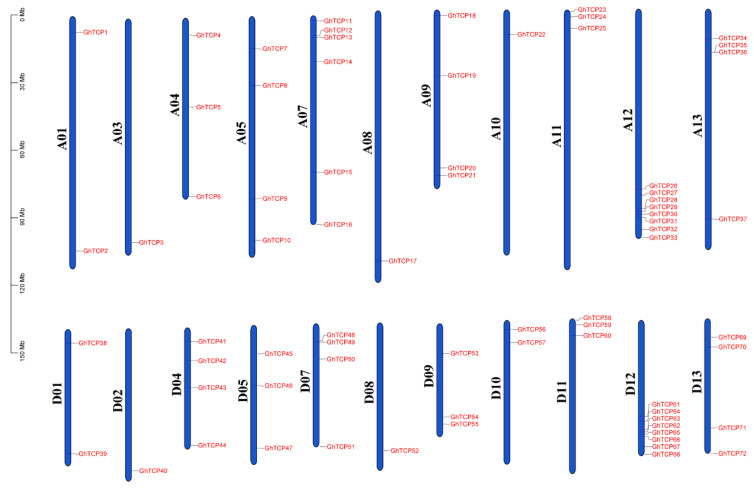
Chromosomal distribution of *TCP* genes of *G. hirsutum*. The scale is in megabases (Mb). Chromosome numbers are indicated at the left of each chromosome.

The genomic annotation file of *G. hirsutum* (ZM24) was used to determine the chromosomal location of the *GhTCP* genes and the distribution on the chromosomes of these genes was visualized (Figure 4). All the *GhTCP* genes were located at 22 of 26 chromosomes. The number of *GhTCP* genes on each chromosome was not uniformly distributed, ranging from 0 to 8. For instance, chromosome A12/D12 contained the most *GhTCP* genes, with a total of eight *GhTCP* genes. However, there no *GhTCP* gene was found on the A02, D03, A06, or D06 chromosomes.

**Figure 5:**
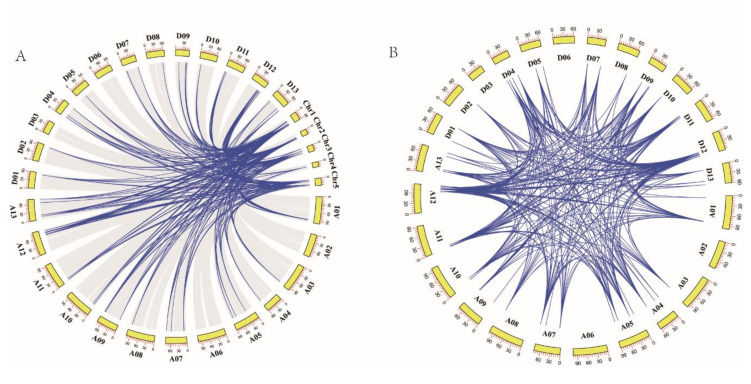
Synteny of *TCP* genes. (**A**) Synteny analysis of *TCP* genes between *G. hirsutum* and *Arabidopsis*. Colored lines indicate the syntenic regions between *G. hirsutum* and *Arabidopsis* chromosomes. (**B**) Chromosomal distribution and synteny analysis of cotton *TCP* genes. The chromosomal positions of cotton *TCP* genes were identified. Colored lines connecting two chromosomal regions indicate the syntenic regions of the cotton genome.

Phylogenetic analysis revealed the existence of a large number of homologous and heterologous gene pairs produced by gene replication. Collinearity analysis between the *GhTCP* genes and the *AtTCP* genes demonstrated that there were 323 pairs of orthologous/paralogous *TCP* genes between *G. hirsutum* and *Arabidopsis* and that there were 167 gene pairs between the A subgenome of *G. hirsutum* and *Arabidopsis*. Similarly, there were 156 gene pairs between the D subgenome of *G. hirsutum* and *Arabidopsis*. Notably, there was fragment duplication between each *AtTCP* gene and 2–4 *GhTCP* genes, indicating that fragment duplication events played an important role in *TCP* gene family expansion during the evolution of *G. hirsutum* (Figure 5A).

The collinear analysis indicated that there were 537 pairs of homologous *TCP* genes in *G. hirsutum*. Specifically, there were 121 gene pairs within the A subgenome of *G. hirsutum*, 155 gene pairs between the A subgenome and D subgenome of *G. hirsutum*, 150 gene pairs between the D subgenome and A subgenome of *G. hirsutum*, and 111 gene pairs in the D genome of *G. hirsutum*. This indicated that gene replication and fragment duplication were the primary cause of gene family expansion in *G. hirsutum* (Figure 5B).

### 3.5. Expression Profiles of TCP Genes in G. hirsutum

The TYPE2 subfamily *AtTCP* genes plays an important role in the formation of lateral branches, which determines bud structure [61]. Lateral branches development plays a crucial role in controlling plants, which is related to plant yield and growth. Therefore, we further analyzed TYPE2 Gh*TCP* genes.

**Figure 6:**
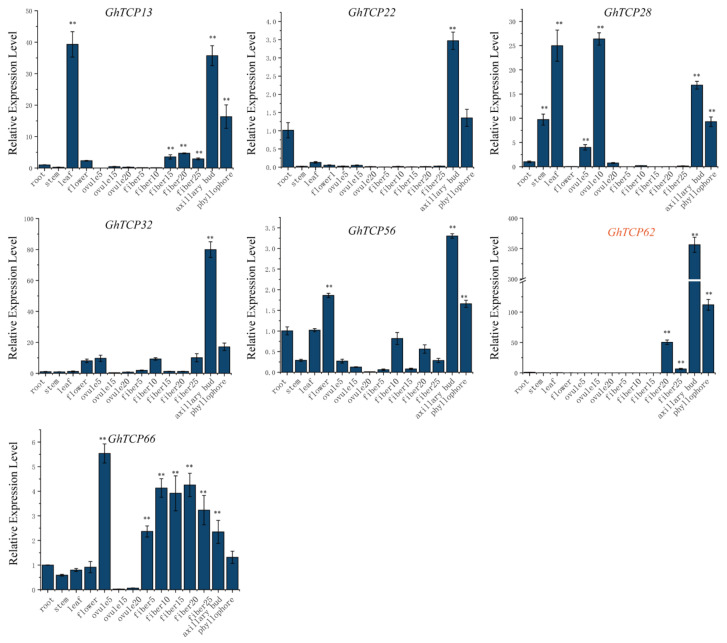
Analysis of tissue-specific gene expression patterns in cotton including root, stem, leaf, flower, fibers and ovules in different stage, axillary bud, and phyllophore. The y-axis represents the expression level of *GhTCP* genes relative to the reference gene Gh*UBQ7*. The error line represents the standard deviation of three repetitions. The asterisks indicated significant differences compared to root (** *p* < 0.01 by *t*-test).

The expression pattern of a gene can be used to predict its function. Therefore, we analyzed the tissue-specific expression profile of TYPE2 subfamily *GhTCP* genes in the root, stem, leaf, flower, ovule, fiber, and other tissues. Our results demonstrated that most *GhTCP* genes, except for *GhTCP66*, were highly expressed in the axillary buds and phyllophore. *GhTCP62* had the highest and most specific expression in the axillary buds and phyllophores. The *GhTCP62* gene belongs to the *TB1* subfamily, which regulates the branches of various plant species. This indicates that *GhTCP62* could regulate branching in *G. hirsutum* (Figure 6).

**Figure 7:**
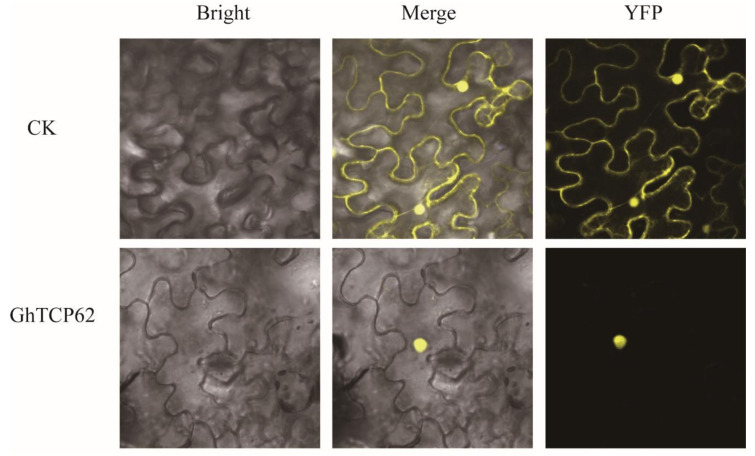
Subcellular localization of *GhTCP62*.

Studies have demonstrated that the localization of the putative nuclear localization signal (KRGK) in the N-terminus of the protein suggests that protein localization occurs in the nucleus [63,64]. We tested the transient expression of *GhTCP62* by injecting *GhTCP62-YFP* into tobacco leaves and found that the *GhTCP62* protein was localized in the nucleus (Figure 7).

### 3.6. Overexpression of GhTCP62 in A. thaliana Inhibits Shoot Branching

**Figure 8:**
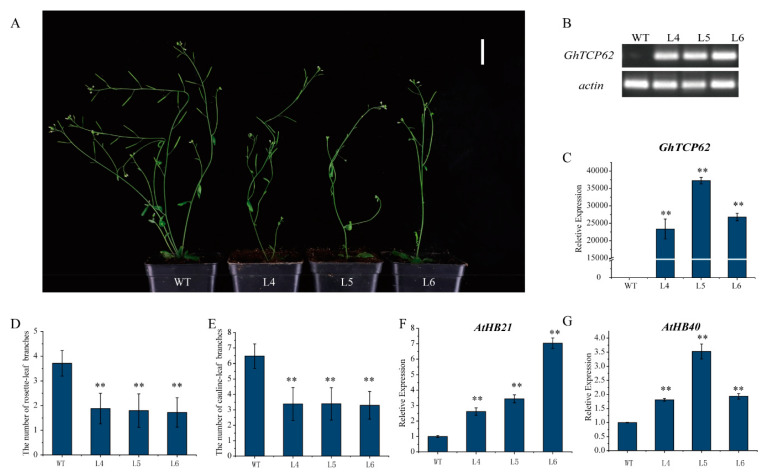
Overexpression of *GhTCP62* in *Arabidopsis* reduced the number of branches. (**A**) Branching phenotypes of 35-day-old WT plants and three overexpressed *35S-GhTCP62* lines. (**B**) Semi-quantitative PCR and (**C**) the expression level of *GhTCP62* in *GhTCP62* overexpressed cell lines was analyzed by qRT-PCR. (**D**) Quantitative analysis of rosette-leaf branches in 35-day-old WT and *GhTCP62* OE lines. (**E**) Quantitative analysis of cauline-leaf branches in 80-day-old WT and *GhTCP62* OE lines. (**F**) The expression level of *AtHB21* in *GhTCP6*2-OE line was analyzed by qRT-PCR. (**G**) The expression level of *AtHB21* in the *GhTCP62*-OE line was analyzed by qRT-PCR. The error line represents the standard deviation of three repetitions. The asterisks indicated significant differences compared to WT (** *p* < 0.01 by *t*-test).

To verify the function of *GhTCP62*, *Arabidopsis*-overexpression (OE) lines were generated using the *GhTCP62* gene. Three homozygous lines (L4, L5, L6) were selected for observation and statistical analysis to further analyze the levels of gene expression and shoot branching. These OE lines had fewer rosette-leaf branches and cauline-leaf branches (Figure 8A). Semi-quantitative PCR and RT-qPCR analysis confirmed high *GhTCP62* gene expression in overexpression lines compared to WT (Figure 8B,C). The *GhTCP62* gene overexpression lines featured fewer cauline-leaf branches and rosette-leaf branches in 35-day-old seedlings compared to the WT (Figure 8A,D,E). The *GhTCP62* gene overexpression lines had only one small or dormant rosette-leaf bud compared to the WT, where rosette-leaf buds developed rosette-branches. The *GhTCP62* gene overexpression lines featured one or two rosette-leaf branches, while three or four rosette branches were observed in the WT line (Figure 8A,D). Fewer cauline-leaf branches (3) were observed in the OE lines, while six were observed in the WT line in 35-day-old plants (Figure 8A,E). In addition, we also selected the functional deletion mutant *brc1-2* to conduct the mutant’s complement experiment, and the results showed that *GhTCP62* could complement the phenotype of *brc1-2* (Figure S1). These results indicate that *GhTCP62* negatively regulates the number and growth vigor of shoot branching.

*BRC1*, a homologous gene of *BRC2*, directly regulates the bud dormancy genes *HB21*, *HB40*, and *HB53* in *Arabidopsis* [65]. In this study, *GhBRC1* and *GhBRC2* are the homologous genes of *Arabidopsis BRC1* and *BRC2*, respectively. The *GhHB21* and *GhHB40* found in *G. hirsutum* indicated that the expression levels of *GhHB21* and *GhHB40* were positively regulated by *GhTCP32* (*GhBRC1*) [64]. *GhTCP62* (*GhBRC2*) and *GhTCP32* (*GhBRC2*) share high homology, leading us to speculate that *GhTCP62* could also regulate bud activity and branching via the *GhHB* genes. The real-time fluorescence quantitative PCR analysis confirmed high expression levels of *HB21* and *HB40* genes in the *GhTCP62* OE lines (Figure 8F,G). These results indicate that *GhTCP62* could regulate bud activity and branching through the *GhHB21* and *GhHB40* genes in *G. hirsutum*, which increases ABA levels and inhibits bud activity [66].

## 4. Discussion

### 4.1. TCP Gene Plays an Important Role in Plants

*TCP* transcription factors are a class of plant-specific transcription factors, which play an important and varied role in plant growth and development [57,58], including branching [61,67], regulating leaf development [68], seed germination [69], and regulating the circadian clock [70]. According to the sequence homology of the *TCP* domain, *TCP* proteins are divided into two classes: class I and class II [71]. According to sequence differences within the *TCP* domain, class I can be further subdivided into two clades, CIN and CYC/TB1 [72].

Different types of *TCP* transcription factors have different functions [57]. Based on mutational studies of multiple members of this subfamily, class II *TCP* members show inhibited plant growth and cell proliferation [73]. The main function of TB1 genes is regulating axillary bud development and branching [58,67]. Studies have demonstrated that *AtTCP12* (*BRC2*) influences shoot branching [67], and that *AtTCP18* (*BRC1*) controls stem branching and interacts with FLOWERING LOCUS T to repress the floral transition of the axillary meristems [61]. The CIN subclade *TCP* genes interfere with several different cellular pathways that control leaf development [58]. For instance, in *Arabidopsis*, four *TCP* genes (*AtTCP3*, *AtTCP4*, *AtTCP10*, and *AtTCP24*) involved in the regulation of leaf development were downregulated in the iamt1-D line, resulting in crinkled leaf phenotypes [74]. The *Arabidopsis* triple mutants (Attcp2, 4, 10-mutants) display epinastic cotyledons and slightly enlarged leaves [75]. Class I (PCF) *TCP* factors primarily induce cell division and promote plant growth [20]. *AtTCP14* activates embryonic growth potential during seed germination, and the *AtTCP14* mutant shows delayed germination, indicating a role in the GA regulation of embryo growth during seed germination [69]. Additionally, leaf developmental traits in the mutants of *AtTCP8*, *AtTCP15*, *AtTCP21*, *AtTCP22*, and *AtTCP23* were altered. Transgenic plants expressing *AtTCP7SRDX* and *AtTCP23SRDX* indicate their role in cell proliferation [76].

### 4.2. Gene Replication Events Are the Main Reason for the Expansion of the TCP Gene Family

In this study, we identified 38 *GrTCPs* in *G. raimondii*, 36 *GaTCP*s in *G. australe*, 72 *GbTCPs* in *G. barbadense*, and 72 *GhTCPs* in *G. hirsutum* (ZM24), and analyzed their basic information. In previous studies, other researchers identified 73 *TCP* genes in *G. hirsutum* (TM-1), which differs from the 72 genes we identified. This result indicates that the *TCP* family differs among different cotton species. The *TCP* family gene that we identified in *G. hirsutum* (ZM24) was twice the size ofthat of *G. arboreum*, which suggests that *G. arboreum* is diploid and *G. hirsutum* (ZM24) is tetraploid. We then analyzed the conserved domain in *G. hirsutum*, and found that Motif1 was present in almost all family members. Different motifs were often present among family members on different branches of the evolutionary tree. These results demonstrate that Motif1 could be a conserved motif of the *TCP* family, while other motifs could exist on specific branches of the evolutionary tree, since different *TCP* genes perform specific functions.

After analyzing the gene structure, we found that most *TCP* genes only contain one exon, indicating that the *TCP* gene family could have emerged and expanded in later stages of evolution. Collinearity analysis of the *TCP* gene family indicated that gene replication events played an important role in the extension of the *TCP* gene family in cotton. In general, the *TCP* gene family could have emerged later in its evolutionary history and expanded its family through gene replication.

### 4.3. GhTCP62 Regulate Shoot Branching in Cotton

Several studies have been conducted to better understand the mechanism of plant branching. These found that many *TCPs* (TEOSINTE BRANCHED1 (TB1) from maize, *Arabidopsis BRC1* and *BRC2*, and rice, PROLIFERATING CELL FACTOR, were involved in plant branching [64,77]. The ectopic overexpression of *OsTB1* significantly reduced lateral branching [78]. Similarly, the overexpression of *BRC1* led slowed the growth of the meristem, slowed bud transformation, and reduced the number of branches [79]. *BRC1-2* deletion mutants accelerated the development of the meristem, induced rapid bud transformation, and increased the number of branches [67].

*BRC2* plays a unique role in the development of axillary buds and shoot branching patterns [61,67]. Pcbrc2-1 knockout lines significantly increased the number of branches compared with the WT [80]. Similarly, *BRC2* RNAi and T-DNA insertion lines slightly enhanced bud growth [67]. In this study, RT-qPCR results demonstrated that *GhTCP62* was specifically expressed at the base of the stems in upland cotton, indicating that *GhTCP62* affected cotton branching. *GhTCP62* is located in the nucleus and features typical transcription factor characteristics, and its overexpression in *Arabidopsis* decreased the number of rosette-leaf branches and cauline-leaf branches. This suggests that *GhTCP62* could regulate cotton branches.

### 4.4. GhTCP62 Regulates Bud Activity and Branching Via HB21 and HB40 Genes

Based on the known upstream gene regulatory network of *BRC1* and the downstream target genes of *BRC1*, some studies have reported the central role of *BRC1* in shoot branching [67,81]. *BRC1* directly regulates the bud dormancy genes *HB21*, *HB4*0, and *HB53* in *Arabidopsis* [65]. The *BRC1* and *HB* genes increase ABA levels and inhibit bud activity [66]. Phylogenetic analysis identified the presence of *GhHB21* and *GhHB40* in cotton, and *GhBRC1* positively regulated the expression of these genes [64]. In this study, the expression levels of *HB21* and *HB40* genes were higher in *GhTCP62* overexpression lines than in WT plants. *GhTCP32* and *GhTCP62* are homologous genes of *BRC1* and *BRC2*, leading us to speculate that *GhTCP62* could also regulate branches via the *HB* gene. This was confirmed by real-time fluorescence quantitative PCR analysis. Along with these results, *GhTCP62* could regulate bud activity and branching through the *HB21* and *HB40* genes, which increase ABA levels and inhibit bud activity [66].

## 5. Conclusions

*TCP* transcription factors play important roles in plant growth and development. In this study, 218 *TCP* genes were identified in four cotton species and were divided into seven subfamilies. We observed similar exon-intron structures and protein motif distribution patterns for *GhTCP* genes. The *GhTCP* genes were unevenly distributed on 24 chromosomes, with fragment replication events. *GhTCP62* was highly expressed in the cotton axillary buds. Furthermore, the overexpression of *GhTCP62* decreased the number of rosette-leaf branches and cauline-leaf branches in *Arabidopsis*. Moreover, the increased expression of *HB21* and *HB40* genes in *Arabidopsis* overexpressing *GhTCP62* suggests that *GhTCP62* may regulate branching by positively regulating *HB21* and *HB40*.

## Data Availability

All datasets generated for this study are included in the article and Supplementary Materials.

## Supplementary Materials

The following are available online at https://www.mdpi.com/article/10.3390/biology10111104/s1. Figure S1: *GhTCP62* complementary *brc1-2* phenotype in *Arabidopsis*. **Table S1**: Characteristics of *AtTCP* family genes and the encoded proteins. Table S2: Characteristics of *GhTCP* family genes and the encoded proteins. Table S3: Primers used in this study.
