# Supplementary material for: Systematic Characterization of TCP Gene Family in Four Cotton Species Revealed That GhTCP62 Regulates Branching in Arabidopsis
Source: Biology (Basel). 2021 Oct 26;10(11):1104. doi: 10.3390/biology10111104 (PMC8614845; doi:10.3390/biology10111104)
Supplement: Supplementary file 1 [file biology-10-01104-s001.zip › biology-1411118-supplementary/TableS1.pdf]

Table S1. Characteristics of *AtTCP* family genes and the encoded proteins.

Gene ID	Rename	Location	polypentide length (aa)	CDS length(bp)	Genome length(bp)
AT1G30210	AtTCP24	Chr1:10627185-10630610	324	975	3425
AT1G35560	AtTCP23	Chr1:13115826-13117235	341	1026	1409
AT1G53230	AtTCP3	Chr1:19849788-19851725	391	1176	1937
AT1G58100	AtTCP8	Chr1:21512384-21514022	401	1206	1638
AT1G67260	AtTCP1	Chr1:25167462-25169307	359	1080	1845
AT1G68800	AtTCP12	Chr1:25847066-25848540	356	1071	1474
AT1G69690	AtTCP15	Chr1:26216224-26217851	325	978	1627
AT1G72010	AtTCP22	Chr1:27107567-27109275	375	1128	1708
AT2G31070	AtTCP10	Chr2:13220478-13222609	361	1086	2131
AT2G37000	AtTCP11	Chr2:15540299-15541248	188	567	949
AT2G45680	AtTCP9	Chr2:18820242-18821889	356	1071	1647
AT3G02150	AtTCP13	Chr3:391057-392758	278	837	1701
AT3G15030	AtTCP4	Chr3:5061681-5064115	420	1263	2434
AT3G18550	AtTCP18	Chr3:6383508-6385663	433	1302	2155
AT3G27010	AtTCP20	Chr3:9957376-9958898	314	945	1522
AT3G45150	AtTCP16	Chr3:16531179-16531676	165	498	497
AT3G47620	AtTCP14	Chr3:17558849-17560767	489	1470	1918
AT4G18390	AtTCP2	Chr4:10162953-10165562	365	1098	2609
AT5G08070	AtTCP17	Chr5:2584885-2585613	242	729	728
AT5G08330	AtTCP21	Chr5:2680744-2681813	239	720	1069
AT5G23280	AtTCP7	Chr5:7842971-7844275	250	753	1304
AT5G41030	AtTCP6	Chr5:16428706-16429437	243	732	731
AT5G51910	AtTCP19	Chr5:21094547-21095848	293	882	1301
AT5G60970	AtTCP5	Chr5:24535570-24537047	360	1083	1477
