# Supplementary material for: Systematic Characterization of TCP Gene Family in Four Cotton Species Revealed That GhTCP62 Regulates Branching in Arabidopsis
Source: Biology (Basel). 2021 Oct 26;10(11):1104. doi: 10.3390/biology10111104 (PMC8614845; doi:10.3390/biology10111104)
Supplement: Supplementary file 1 [file biology-10-01104-s001.zip › biology-1411118-supplementary/TableS2.pdf]

Table S2. Characteristics of *GhTCP* family genes and the encoded proteins.

Gene ID	Rename	Location	polypentide length (aa)	CDS length(bp)	Genome length(bp)
Ghicr24_A01G057400.1	GhTCP1	A01:7023145-7026280	446	1341	3136
Ghicr24_A01G202500.1	GhTCP2	A01:104154003-104156434	550	1653	2432
Ghicr24_A03G191300.1	GhTCP3	A03:99313669-99316451	258	777	2783
Ghicr24_A04G042900.2	GhTCP4	A04:7562235-7564791	451	1356	2557
Ghicr24_A04G072300.1	GhTCP5	A04:39545909-39548274	356	1071	2366
Ghicr24_A04G157400.1	GhTCP6	A04:79281420-79284235	487	1464	2816
Ghicr24_A05G152300.6	GhTCP7	A05:14322728-14327175	410	1233	4448
Ghicr24_A05G283300.1	GhTCP8	A05:30684057-30686044	422	1269	1988
Ghicr24_A05G361500.1	GhTCP9	A05:80917951-80919677	300	903	1727
Ghicr24_A05G400600.1	GhTCP10	A05:99460765-99462510	309	930	1746
Ghicr24_A07G020800.1	GhTCP11	A07:2351694-2355457	366	1101	3764
Ghicr24_A07G070500.1	GhTCP12	A07:8912951-8914751	418	1257	1801
Ghicr24_A07G074400.1	GhTCP13	A07:9575666-9576881	266	801	1216
Ghicr24_A07G120800.1	GhTCP14	A07:20495291-20498685	388	1167	3395
Ghicr24_A07G190700.1	GhTCP15	A07:69538146-69541601	395	1188	3456
Ghicr24_A07G257300.1	GhTCP16	A07:92783070-92784436	304	915	1367
Ghicr24_A08G196200.1	GhTCP17	A08:111177210-111178244	312	939	1035
Ghicr24_A09G010000.1	GhTCP18	A09:2483362-2485365	285	858	2004
Ghicr24_A09G055400.1	GhTCP19	A09:29157373-29159195	298	897	1823
Ghicr24_A09G169000.1	GhTCP20	A09:70235172-70236132	204	615	961
Ghicr24_A09G200000.1	GhTCP21	A09:73505310-73506982	341	1026	1673
Ghicr24_A10G074100.1	GhTCP22	A10:10883098-10884698	301	906	1601
Ghicr24_A11G006700.1	GhTCP23	A11:667771-671530	386	1161	3760
Ghicr24_A11G035600.1	GhTCP24	A11:3030789-3031976	395	1188	1188
Ghicr24_A11G090800.1	GhTCP25	A11:8150412-8151428	338	1017	1017
Ghicr24_A12G148100.1	GhTCP26	A12:80029742-80030473	243	732	732
Ghicr24_A12G159200.2	GhTCP27	A12:82675554-82677225	300	903	1672
Ghicr24_A12G185500.1	GhTCP28	A12:88654121-88655799	344	1035	1679
Ghicr24_A12G190700.1	GhTCP29	A12:89834493-89836050	501	1506	1558
Ghicr24_A12G196700.1	GhTCP30	A12:91053274-91054494	406	1221	1221
Ghicr24_A12G202800.1	GhTCP31	A12:92500084-92502383	325	978	2300
Ghicr24_A12G245700.1	GhTCP32	A12:97775752-97776906	384	1155	1155
Ghicr24_A12G288000.1	GhTCP33	A12:101555903-101562085	355	1068	6183
Ghicr24_A13G066900.1	GhTCP34	A13:13134103-13134870	255	768	768
Ghicr24_A13G082500.1	GhTCP35	A13:19158117-19159169	350	1053	1053
Ghicr24_A13G082600.1	GhTCP36	A13:19164318-19165412	364	1095	1095
Ghicr24_A13G172200.1	GhTCP37	A13:93258959-93261131	409	1230	2173
Ghicr24_D01G052800.1	GhTCP38	D01:6062642-6065803	444	1335	3162
Ghicr24_D01G200300.1	GhTCP39	D01:55192837-55195846	548	1647	3010
Ghicr24_D02G207500.1	GhTCP40	D02:63162055-63162828	257	774	774
Ghicr24_D04G042600.1	GhTCP41	D04:6050254-6051195	313	942	942

Ghicr24_D04G080700.1	GhTCP42	D04:14655520-14657387	300	903	1868
Ghicr24_D04G099500.1	GhTCP43	D04:26572897-26575448	435	1308	2552
Ghicr24_D04G195400.1	GhTCP44	D04:52208526-52210537	487	1464	2012
Ghicr24_D05G146600.2	GhTCP45	D05:12620398-12626674	410	1233	6277
Ghicr24_D05G274500.1	GhTCP46	D05:26823622-26824887	421	1266	1266
Ghicr24_D05G346900.1	GhTCP47	D05:54523969-54526367	401	1206	2399
Ghicr24_D07G067700.1	GhTCP48	D07:7530959-7532659	409	1230	1701
Ghicr24_D07G071800.1	GhTCP49	D07:8171899-8173478	266	801	1580
Ghicr24_D07G115000.1	GhTCP50	D07:15745445-15748909	350	1053	3465
Ghicr24_D07G246700.1	GhTCP51	D07:54662671-54663906	302	909	1236
Ghicr24_D08G198600.1	GhTCP52	D08:56794383-56795419	318	957	1037
Ghicr24_D09G043200.1	GhTCP53	D09:13156825-13157922	301	906	1098
Ghicr24_D09G157600.1	GhTCP54	D09:41434272-41435281	232	699	1010
Ghicr24_D09G188300.1	GhTCP55	D09:44574659-44575672	337	1014	1014
Ghicr24_D10G043700.1	GhTCP56	D10:3986012-3991565	463	1392	5554
Ghicr24_D10G081300.1	GhTCP57	D10:9830657-9832296	300	903	1640
Ghicr24_D11G006500.1	GhTCP58	D11:617823-621175	346	1041	3353
Ghicr24_D11G034300.1	GhTCP59	D11:2742436-2743623	395	1188	1188
Ghicr24_D11G088400.1	GhTCP60	D11:7476203-7478191	388	1167	1989
Ghicr24_D12G146600.1	GhTCP61	D12:42726925-42728954	243	732	2030
Ghicr24_D12G184800.1	GhTCP62	D12:44659062-44660731	501	1506	1670
Ghicr24_D12G179500.1	GhTCP63	D12:48634246-48636071	344	1035	1826
Ghicr24_D12G156000.1	GhTCP64	D12:49514431-49515936	294	885	1506
Ghicr24_D12G191100.1	GhTCP65	D12:50478444-50479646	400	1203	1203
Ghicr24_D12G198500.1	GhTCP66	D12:51375400-51377845	327	984	2446
Ghicr24_D12G242300.1	GhTCP67	D12:56113339-56114943	385	1158	1605
Ghicr24_D12G279000.1	GhTCP68	D12:59483131-59487021	381	1146	3891
Ghicr24_D13G061200.1	GhTCP69	D13:8275572-8276342	256	771	771
Ghicr24_D13G076400.1	GhTCP70	D13:12525881-12532740	351	1056	6860
Ghicr24_D13G164900.2	GhTCP71	D13:48494332-48496459	409	1230	2128
Ghicr24_D13G252200.1	GhTCP72	D13:59863058-59863648	196	591	591
