# Supplementary material for: Systematic Characterization of TCP Gene Family in Four Cotton Species Revealed That GhTCP62 Regulates Branching in Arabidopsis
Source: Biology (Basel). 2021 Oct 26;10(11):1104. doi: 10.3390/biology10111104 (PMC8614845; doi:10.3390/biology10111104)
Supplement: Supplementary file 1 [file biology-10-01104-s001.zip › biology-1411118-supplementary/TableS3.pdf]

Table S1 Primers used in PCR.

Gene	Primer	Primer pairs (5′–3′)
GhTCP62	Forward	ATGTTTCCTTCCAGCAACAGT
	Reverse	TCATAAGTAAAAATGGTGGAA
35S::GhTCP62-GFP	Forward	ACTCTTGAGGATCCGAATTCATGTTTCCTTCCAGCAACAGT
	Reverse	TCGACAGATCCCCGGGTACCTAAGTAAAAATGGTGGAA
35S::GhTCP62-YFP	Forward	ATTTACGAACGATATCTAGAATGTTTCCTTCCAGCAACAGTTACA
	Reverse	ACCATGTTAATTAAGGATCCTAAGTAAAAATGGTGGAATTTA
35S-GhBRC2-GFP	Forward	ACTCTTGAGGATCCGAATTCATGTTTCCTTCCAGCAACAGT
	Reverse	TCGACAGATCCCCGGGTACCTAAGTAAAAATGGTGGAA
35S-GhBRC2-YFP	Forward	ATTTACGAACGATATCTAGAATGTTTCCTTCCAGCAACAGTTACA
	Reverse	ACCATGTTAATTAAGGATCCTAAGTAAAAATGGTGGAATTTA
ubq7	Forward	AGAGGTCGAGTCTTCGGACA
	Reverse	GCTTGATCTTCTTGGGCTTG
actin	Forward	TGGTGTCAGGTTGGGATGG

	Reverse	CGTGAGAAGAACAGGGTGCT
qGhBIN2	Forward	CTGTTGTTGACCGGAACGATC
	Reverse	CCGTTTCCAAACATTTTGCCTG
qGhBRC2	Forward	ATACTGCGGACTCGGACTCGAT
	Reverse	TGACGATCTTGTGATGCCCAGG
qGhTCP13	Forward	GCAGTGGCAATAACGGCTTTCC
	Reverse	CAGCACAAAGCGTAGAGTGTGC
qGhTCP22	Forward	TTTCGTTCACAGAGACAGCCCC
	Reverse	GCGTTGTTGTTGTTGTTGGTTCC
qGhTCP28	Forward	GGCATCACAAGATCGTCAGCCA
	Reverse	TGCTCTTGCTCATTAGGGTCCG
qGhTCP32	Forward	AGGGAAAAGGCAAGAGCAAGGG
	Reverse	TTGTTGTGGTGGTGGTGTTGGA
qGhTCP56	Forward	GAACCGTGGAATGGCTACTCGT

	Reverse	CACCACTTCGGTCTCGGATGTC
qGhTCP66	Forward	AGGGAAAAGGCAAGAGCAAGGG
	Reverse	TTGTTGTGGTGGTGCTGTTGGA
