# Supplementary figures and images for: Systematic Characterization of TCP Gene Family in Four Cotton Species Revealed That GhTCP62 Regulates Branching in Arabidopsis
Source: Biology (Basel). 2021 Oct 26;10(11):1104. doi: 10.3390/biology10111104 (PMC8614845; doi:10.3390/biology10111104)

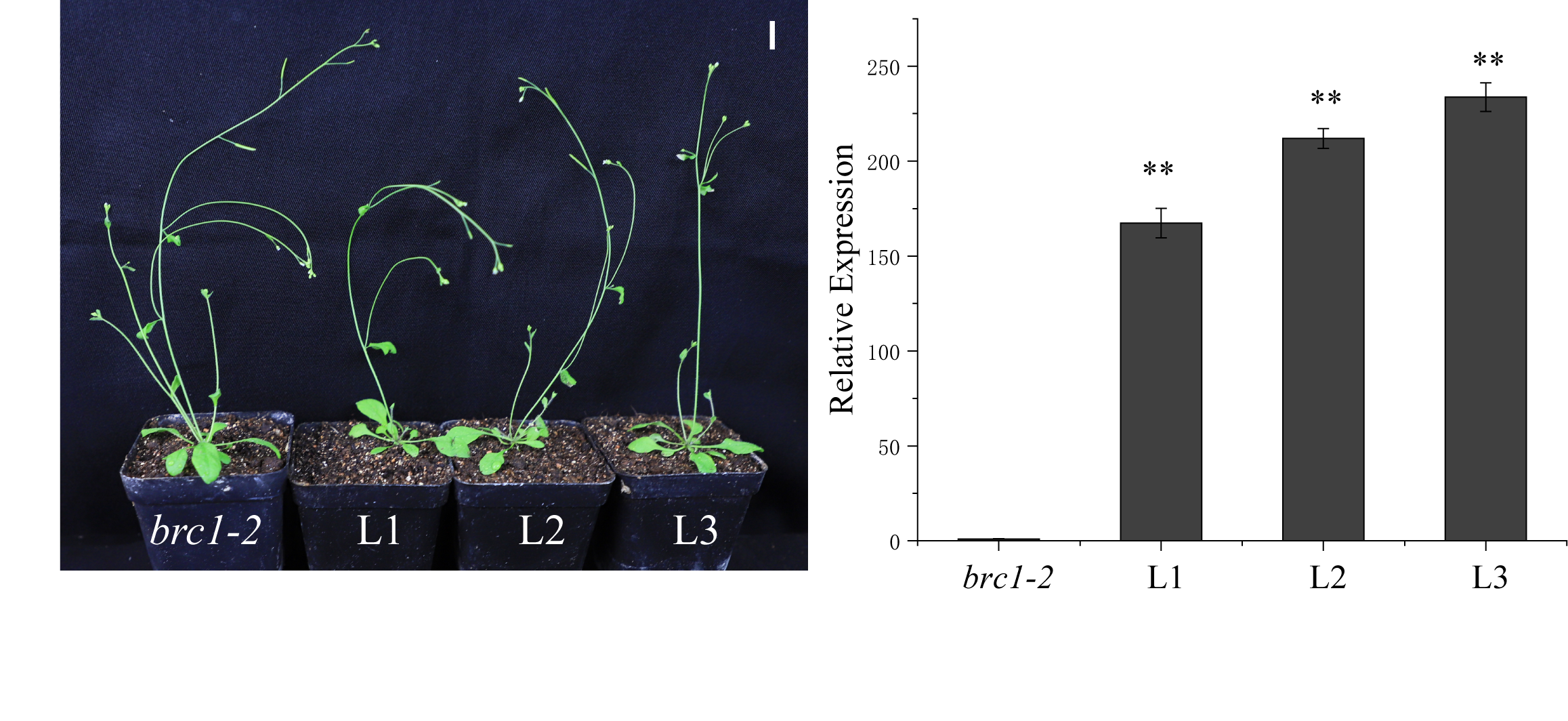

Supplement: Supplementary file 1 [file biology-10-01104-s001.zip › biology-1411118-supplementary/Figure S1.tif]
